# Multiple primary lung adenocarcinomas pre‐operatively diagnosed by discordant epidermal growth factor receptor mutations

**DOI:** 10.1002/rcr2.434

**Published:** 2019-05-20

**Authors:** Yuki Katayama, Sayaka Kawai, Aya Miyagawa‐Hayashino, Yoshizumi Takemura

**Affiliations:** ^1^ Department of Pulmonary Medicine Kyoto Kuramaguchi Medical Center Kyoto Japan; ^2^ Department of Surgical Pathology Kyoto Prefectural University of Medicine Kyoto Japan

**Keywords:** Adenocarcinoma, epidermal growth factor receptor mutation, simultaneous multiple lung cancer, transbronchial biopsy

## Abstract

A 49‐year‐old woman with an abnormal shadow on her chest X‐ray visited our hospital. Chest computed tomography revealed a 13‐mm diameter nodule in S9 on the right and a 47‐mm diameter mass in segment (S) 1 + 2 on the left. She underwent transbronchial biopsy, which revealed that both tumours had the same histology of papillary adenocarcinoma. The indications of radical surgery differ between metastatic and multiple primary cancers; however, the epidermal growth factor receptor mutation screenings turned out to be discordant, with exon 19 deletion in the right and exon 21 L858R mutation in the left tumour. This is the first case report of a pre‐operative diagnosis of multiple primary adenocarcinomas eligible for radical surgery. Thorough assessment is recommended in cases wherein the differential diagnosis is considered to be a factor for surgical indication. Genetic screening provides additional diagnostic information despite the presence of tumours manifesting the same histological type.

## Introduction

Synchronous multiple lung cancers account for approximately 0.5–1.6% of total lung cancers, and their rate has been increasing recently presumably because of the availability of high‐resolution thoracic imaging and the growing incidence of adenocarcinomas among non‐small‐cell lung cancers. However, in clinical practice, it can be complicated to differentiate multiple primary lung cancers from pulmonary metastases prior to surgery. When we encounter patients with multiple pulmonary lesions, there are limitations in both the sampling technique and pathological assessment in determining to which type of cancer they belong. Here, we describe a case of simultaneous multiple bilateral lung adenocarcinomas diagnosed by discordant epidermal growth factor receptor (EGFR) mutations in transbronchial lung biopsy specimens that led to a successful radical surgery.

## Case Report

**Figure 1:**
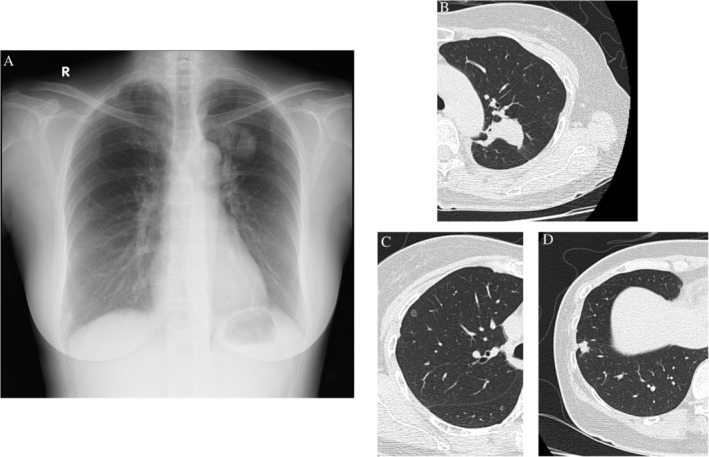
Chest X‐ray and computed tomography (CT). A chest X‐ray revealed a 4‐cm diameter mass in the left upper field. B chest CT revealed a 47‐mm diameter mass in the left segment (S) 1 + 2, C a 6‐mm diameter pure ground‐glass opacity in the right S3, and D an 11‐mm diameter solid nodule in the right S9.

**Figure 2:**
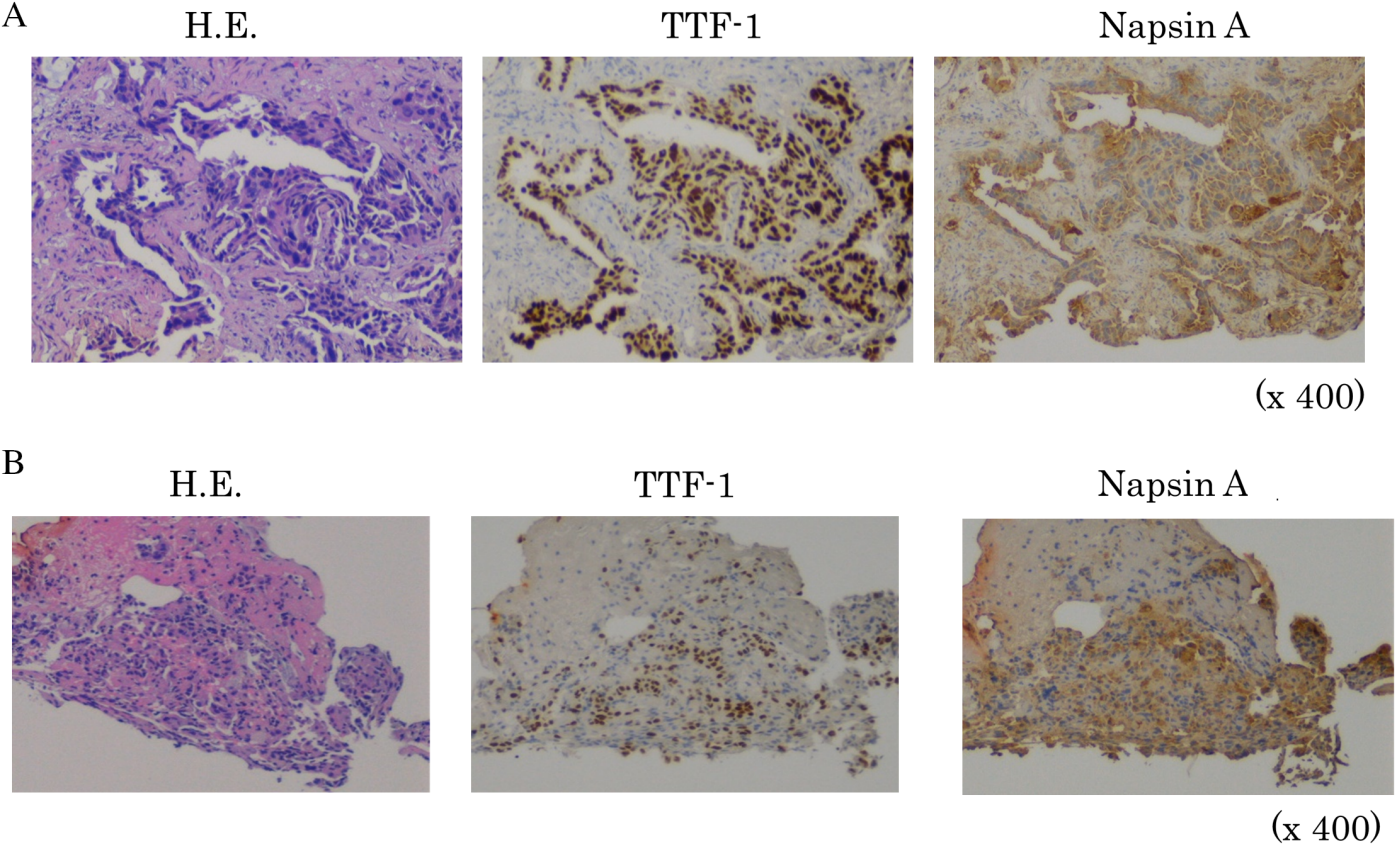
Pathological findings and epidermal growth factor receptor (EGFR) mutation status of the two tumours sampled by bronchoscopy. (A) left upper lobe tumour. Haematoxylin–eosin (HE) staining; papillary adenocarcinoma. Thyroid transcription factor 1 (TTF‐1)‐ and napsin A‐positive, EGFR exon 21 L858R (+). (B) right lower lobe tumour. HE staining; papillary adenocarcinoma, TTF‐1‐ and napsin A‐positive, EGFR exon 19 deletion (+).

A 49‐year‐old woman without a smoking history was referred to our hospital for a detailed examination of an abnormality detected by routine chest X‐ray. The patient was asymptomatic; however, her chest computed tomography (CT) revealed a right upper lobe (RUL) pure ground‐glass nodule (GGN) in segment (S) 3, a 13‐mm diameter right lower lobe (RLL) nodule in S9, and a 47‐mm diameter left upper lobe (LUL) mass in S1 + 2 invading S6 across the interlobar pleura, without enlarged bilateral mediastinal lymph nodes (Fig. 1). A positron emission tomography‐CT (PET‐CT) scan showed maximum standardized uptake values of 2.3 and 6.8 in the RLL and LUL lesions, respectively. Brain contrast‐enhanced magnetic resonance imaging and PET‐CT did not detect any metastatic lesions, including mediastinal lymph node metastases. The whole‐body examination showed that there was no tumourous lesion other than the RUL‐pure GGN, RLL nodule, and LUL mass. Laboratory screening of specific tumour markers, such as carcinoembryonic antigen (1.4 ng/mL), cytokeratin fragment (1.5 ng/mL), and progastrin‐releasing peptide (58.0 pg/mL), did not yield significant results. We performed transbronchial biopsy under X‐ray fluoroscopy guidance for the LUL mass and biopsy for the RLL nodule using endobronchial ultrasonography with the guide sheath method. We obtained an adequate amount of tissues for evaluation; however, the pathological findings of the two tissues (RLL nodule and LUL mass) indicated the same type of papillary adenocarcinoma (thyroid transcription factor 1‐ and napsin A‐positive), making it impossible to distinguish the advanced‐stage (stage IV) lung cancer from the surgery‐eligible multiple lung cancers. However, the EGFR mutation screenings of the two samples demonstrated discordant positive exon 21 L858R mutation in the LUL lesion and positive exon 19 deletion in the RLL lesion; thus, a diagnosis of simultaneous multiple lung adenocarcinomas indicated for surgery (left lung adenocarcinoma cT2bN0M0‐cStage IIA + right lung adenocarcinoma cT1bN0M0‐cStage IA2) was made (Fig. 2).

**Figure 3:**
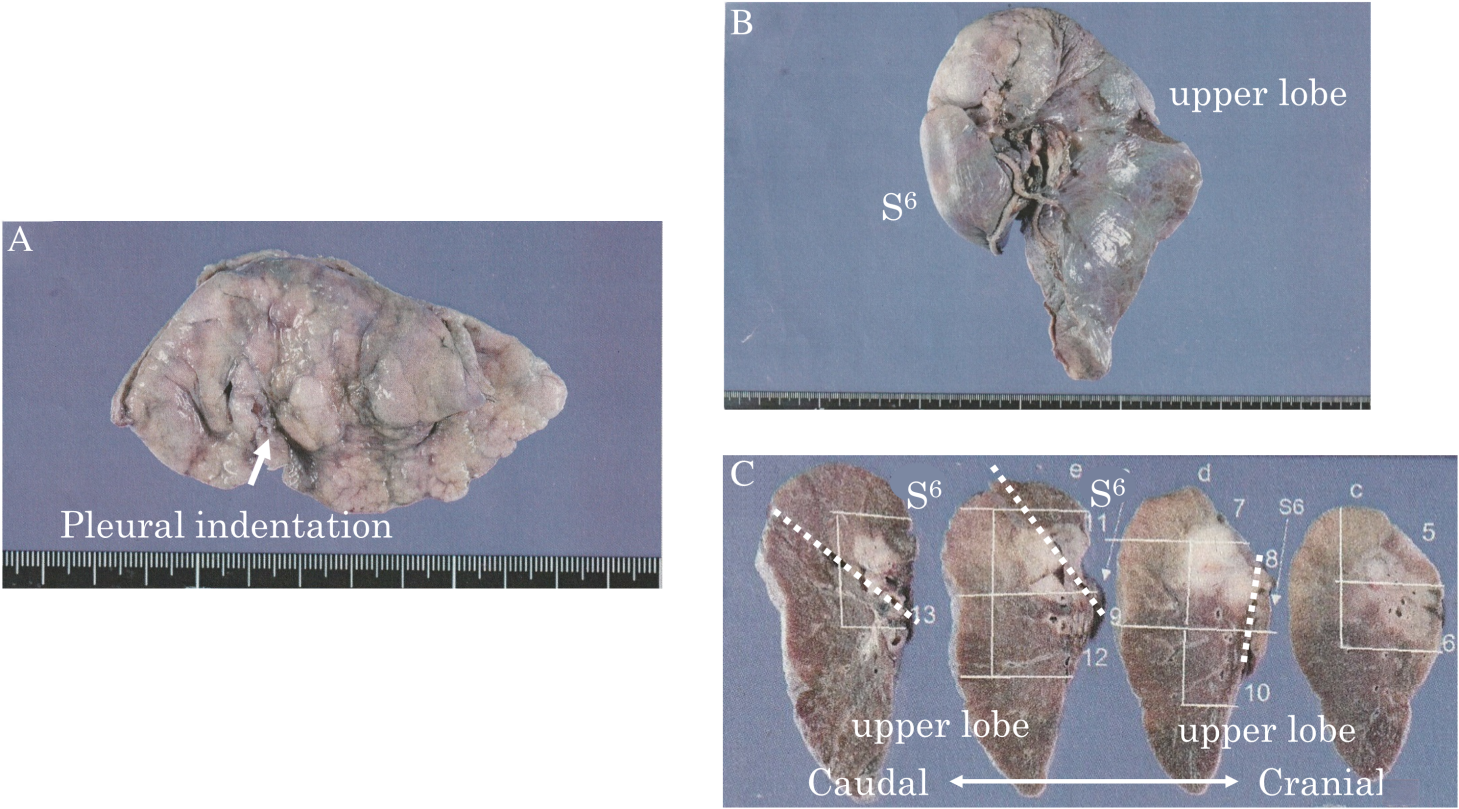
Macroscopic findings of the resected specimens. (A) Partial resection of the right lower lobe. Pleural indentation by the right segment (S) 9 tumour was identical, but the visceral pleura was intact. (B, C) resection of the upper left lobe together with the S6 area. invasion of the left S1 + 2 tumour into the S6 area was observed but not exposed externally or disseminated. the white dotted line indicates the fissure line between the upper and lower lobes.

We referred the patient to a thoracic surgeon in the neighbouring hospital, and she underwent partial resection of the RLL and resection of the LUL together with the S6 area plus node dissection (2a‐1) as the primary surgery. The surgical time was 5 h and 2 min. The macroscopic findings of the resected specimens were as follows: pleural indentation by the RLL tumour was identical; however, the visceral pleura was intact (Fig. 3A). Invasion of the LUL tumour into the S6 area was observed; however, it was not exposed externally, and the visceral pleura was intact (Fig. 3B, C). The final pathological diagnosis and staging of the LUL tumour was invasive micropapillary predominant adenocarcinoma (pT2bpN0) and that of the RLL tumour was invasive papillary adenocarcinoma (pT1bpNx). EGFR mutation screening results with surgically resected specimens were also discordant and compatible with those we obtained before surgery. The post‐operative course was uneventful, and the patient was discharged 16 days after the surgery with post‐operative adjuvant chemotherapy comprising oral tegafur/uracil and scheduled follow‐up of the remaining pure GGN in the right RUL.

## Discussion

Multiple primary lung cancers are occasionally difficult to differentiate from pulmonary metastasis, especially before surgery despite using the best sampling techniques or pathological assessment 1. The general rule of the Tumour, Node, Metastasis Classification states that if there is doubt concerning the correct T, N, or M category to which a particular case should be allotted, the lower category should be chosen 2. It seems reasonable to follow this guideline in actual clinical practice so that patients can choose from more potentially curative treatment options as there tends to be fewer options for patients with an advanced stage of disease. Therefore, radical surgery might have been a recommended option as a therapeutic diagnosis in this case even if differential diagnosis was unrealistic.

However, some matters should be considered prior to performing surgery for undiagnosed patients. Although surgical treatments are not considered to be beneficial for pathologically confirmed contralateral lung metastases or ipsilateral lung metastases 3, they should be performed for eligible multiple primary cancers. Therefore, carefully differentiating multiple primary lung cancers from pulmonary metastasis prior to surgery is of prime importance to prevent conflicting results.

The clinical criteria proposed by Martini and Melamed are widely used in the diagnosis of multiple lung cancers. However, their conventional criteria based on tumour characteristics are known to have potential limitations and inconsistent cases. Cases that met the criteria for intrapulmonary or haematogenous metastases but turned out to be cases of multiple primary cancers have been reported, and were mostly diagnosed by molecular techniques. Therefore, in future, diagnosis should be performed considering the results of genomic analysis. Owing to the widespread advancements in molecular biology regarding diagnostic techniques for lung cancer, genomic analysis can now provide us with additional diagnostic information despite having the same pathological findings 4. In chemo‐naïve conditions, the discordance among EGFR mutations in primary cancers and metastatic lesions (heterogeneity) has been reported to be extremely rare; thus, EGFR mutation screening is useful for differentiating multiple primary lung cancers from pulmonary metastases 5. Additionally, other driver gene mutations provide us with useful information; however, a careful interpretation of the respective false‐negative rates and positive latencies of the mutations is required 4.

We reviewed the literature on simultaneous multiple lung cancers treated by radical surgery and found that all of the cases available to date were diagnosed post‐operatively by analysing surgically resected specimens. Therefore, this is the first case report of a simultaneous multiple lung cancer diagnosed prior to surgery.

In recent years, sampling technique has improved dramatically with the emergence of ultrasonic bronchial endoscopy. A genetic panel examination based on “Cancer Genome Medicine” also will be available in the near future; therefore, we expect more patients with simultaneous multiple lung cancers to be diagnosed prior to surgery.

In conclusion, EGFR mutation screening is useful for distinguishing lung metastasis prior to surgery in cases of simultaneous multiple lung adenocarcinomas. A proactive examination is recommended when diagnosing multiple lung nodules for treatment selection.

### Disclosure Statement

Appropriate written informed consent was obtained for publication of this case report and accompanying images.
